# Efficacy and Safety of Adding Olanzapine to the Standard Preventive Regimen for Chemotherapy-induced Nausea and Vomiting in Children: A Randomized Double-blind Controlled Trial

**DOI:** 10.22037/ijpr.2019.112514.13803

**Published:** 2021

**Authors:** Mona Moshayedi, Ebrahim Salehifar, Hossein Karami, Narjes Hendouei, Mahmoud Mousazadeh, Somaye Alizadeh Haji

**Affiliations:** a *Student Research Committee, Pharmaceutical Sciences Research Center, Hemoglobinopathy Institute, Mazandaran University of Medical Sciences, Sari, Iran. *; b *Pharmaceutical Sciences Research Center, Hemoglobinopathy Institute, Department of Clinical Pharmacy, Faculty of Pharmacy, Mazandaran University of Medical Sciences, Sari, Iran. *; c *Thalassemia Research Center, Faculty of Medicine, Mazandaran University of Medical Sciences, Sari, Iran. *; d *Psychiatry and Behavioral Sciences Research Center, Addiction Institute, Department of Clinical Pharmacy, Faculty of Pharmacy, Mazandaran University of Medical Sciences, Sari, Iran. *; e *Health Sciences Research Center, Addiction Institute, Mazandaran University of Medical Sciences, Sari, Iran. *; f *Thalassemia Research Center, Mazandaran University of Medical Sciences, Sari, Iran.*

**Keywords:** Chemotherapy, Nausea, Vomiting, Olanzapine, Pediatrics, Adverse effects

## Abstract

This study aimed to assess the additive value of olanzapine to a combination of ondansetron and dexamethasone to prevent chemotherapy-induced nausea and vomiting (CINV) in pediatric patients. A total of 40 patients between 4 to 18 years of age were enrolled in this randomized clinical trial. Both groups received a combination of ondansetron and dexamethasone, and 0.14 mg/kg olanzapine or matched placebo were administered for olanzapine and control groups, respectively. The primary end points were complete response and lack of nausea as far as three days after chemotherapy evaluated by the Common Terminology Criteria for Adverse Effects (CTCAE) v5.0 and the Multinational Association of Supportive Care in Cancer (MASCC) Anti-emesis Tool (MAT). Side effects of olanzapine were also analyzed. In patients receiving the standard regimen of ondansetron and dexamethasone, nausea was observed in 10.5% and 21% of patients according to MAT and CTCAE scales, respectively. In the olanzapine group, 37.5% (MAT scale) and 31.3% (CTCAE scale) of patients developed nausea. Complete response was observed in 84% (MAT scale) and 94.7% (CTCAE scale) of patients in the placebo group receiving ondansetron and dexamethasone. In comparison, it was observed in 87.5% (MAT scale) and 81.25% (CTCAE scale) for patients allocated to the olanzapine group. Neither acute nor delayed CINV was statistically different between placebo and olanzapine groups. The frequency of adverse effects was higher in the olanzapine group. Adding olanzapine to the standard regimen of CINV prophylaxis was only unhelpful in pediatric patients receiving moderately emetogenic chemotherapy but also associated with a higher rate of minor side effects.

## Introduction

Chemotherapy-induced nausea and vomiting (CINV) is one of the most common and distressing side effects of chemotherapy (1). It is defined as acute CINV, delayed CINV or anticipatory CINV. Acute CINV occurs within 24 h after chemotherapy, while delayed CINV begins 24 h or more after chemotherapy and can last up to several days after chemotherapy infusion is completed (2). The release of various neurotransmitters leads to different phases of CINV (3). Acute CINV is mainly caused by a release of serotonin from enteric cells and is thus efficiently controlled by 5-HT3 receptor inhibitors such as ondansetron (4, 5). Delayed CINV is caused by other mechanisms, including activation of neurokinin-1 (NK-1) receptors by substance P. Identification of this mechanism has led to the development of NK-1 receptor inhibitor (*e.g.*, aprepitant) which was effective in preventing delayed CINV (6). Corticosteroids and dopamine receptor antagonists are other agents approved for CINV prophylaxis (7). 

The pediatric population is more vulnerable to CINV, albeit the use of novel anti-emetics in pediatric oncology is less studied due to safety concerns (5). CINV in children has a significant impact on the quality of life and may also lead to life-threatening complications such as respiratory aspiration and prolonged hospitalization (8). Thus, studies are underway to define effective guidelines to control CINV in pediatric patients and adapt the protocols used in adult patients for the pediatric population (9). 

Olanzapine is a dopamine receptor antagonist approved for the treatment of psychotic disorders and has been especially effective in treating breakthrough and refractory CINV in adults (10). In the adult population, olanzapine has been shown to potentiate the preventive effects of 5-HT3 inhibitors and corticosteroids in acute and delayed CINV (11). Other studies have demonstrated olanzapine to be as effective as aprepitantin providing a more cost-effective therapy option (12). It has an acceptable safety profile with no grade 3 or 4 toxicities (13). 

Although studies provide evidence for the safety and efficacy of olanzapine in the successful prevention of CINV in adults, similar research is limited in children (14). This study aimed to determine the safety and efficacy of adding olanzapine to the standard anti-emetic regimen of dexamethasone and ondansetron in children of 4 to 18 years of age receiving moderately emetogenic chemotherapies. 

## Experimental

*Design and patients*

This randomized double-blinded clinical trial was conducted in the Pediatric Oncology Division of Boo’AliSina hospital affiliated to the Mazandaran University of Medical Sciences from June 2017 to May 2019. The study design was compliant with the Declaration of Helsinki, approved by the institutional review board (IR.MAZUMS.REC.1397.1256), and registered in the Iranian Registry of Clinical Trial (IRCT20090613002027N14). Those administering medication and those assessing nausea and vomiting or adverse effects were blinded in this study.

Subjects were children between 4 and 18 years of age who were receiving chemotherapy with a moderate risk of emetogenicity, according to the Chemotherapy Oncology Group (COG) guideline 2015 (15). All the enrolled subjects were administered ondansetron and dexamethasone as the standard regimen for preventing acute CINV in moderately emetogenic chemotherapies (15, 16). Exclusion criteria were children below 14 kg of weight, serum bilirubin above 3 mg/dL, brain tumor diagnosis, uncontrolled high blood pressure, and receipt of medications carrying the risk of inducing neuroleptic malignant syndrome within 30 days before the study. Patients who received olanzapine or other antipsychotics, amifostine, inducers and inhibitors of CYP1A2 and quinolone antibiotics within 15 days before enrollment were also excluded. 

*Procedures*

Patients were enrolled in the study after signing the written informed consent by their parents. Patients were randomly assigned to receive olanzapine or placebo according to Permuted block randomization method with 4 cases in each block. The standard CINV prophylactic treatment (*i.e.*, ondansetron and dexamethasone) were administered for all patients. Both olanzapine and placebo were made by Bakhtar Biochimi Pharmaceutical Company (Sanandaj, Iran). Ondansetron was given at a dose of 0.15 mg/kg/dose intravenously (IV) at inpatient setting, or the same dose orally (PO) at outpatient setting, 30 min before chemotherapy and then every 8 h until 24 h after termination of chemotherapy. The patients also received dexamethasone (2 mg/dose for body surface area of less than or equal to 0.6 m^2^ and 4 mg/dose for body surface area of more than 0.6 m^2^) IV 30 min before chemotherapy or PO 1 h prior, and then every 12 h until 24 h after termination of chemotherapy. Olanzapine was given 0.14 mg/kg/dose (maximally 10 mg per day). If necessary, the dose was rounded to the nearest dose of 2.5 mg as the starting dose, given orally 1 h prior to chemotherapy, and then given once daily until 72 h after termination of chemotherapy to serve as prophylactic for delayed CINV. Administration of medications to treat CINV, including olanzapine or placebo was in the hospital and therefore, adherence was ensured in enrolled patients.

The duration of chemotherapy was 1 to 3 days, according to the agent being administered. The risk of emetogenicity of the chemotherapy agent was extracted based on chemotherapy oncology group (COG) guideline 2015 (15). 

The primary outcomes of the study were complete response (no vomiting and no need for rescue therapy) and lack of nausea up to 3 days after completion of chemotherapy. CINV was evaluated using two scoring systems, the Multinational Association for Supportive Care in Cancer (MASCC) Anti-emesis Tool (MAT) and the Common Toxicity Criteria for Adverse Events (CTCAE) grading system (17, 18). The frequency of nausea or vomiting experienced per day for three days after the completion of chemotherapy was reported by patients and their parents and collected by trained medical staff based on the MAT and CTCAE questionnaires. To assess the safety of olanzapine the secondary outcome, weight (kg), serum fasting blood sugar (FBS), prolactin, triglyceride, cholesterol, alanine aminotransferase (ALT) and aspartate aminotransferase (AST), as well as systolic and diastolic blood pressures were measured at baseline by trained medical staff as well as three days after completion of chemotherapy. Extrapyramidal symptoms were evaluated according to the Simpson Angus Scale (SAS), Barnes Akathisia Scale (BAR) and Abnormal Involuntary Movement Scale (AIMS) scales (19). 

*Statistics*

Using two sample ratio comparisons in G-power software a sample size of 40 would lead to a power of 80% with a 95% confidence interval to evaluate efficacy. The SPSS software version 24.0 (SPSS, Inc., Chicago, IL) was applied for statistical analysis. The normality of data was checked with Shapiro-Wilk Test. Mann-Whitney U test (comparison of continuous variables between two groups), Wilcoxon matched-pair signed-rank test (comparison of continuous variables before and after treatment), and Chi^2^ test (comparing the qualitative data including the incidence of nausea, vomiting and side effects between placebo and olanzapine groups) were used for analysis. The method of analysis was per-protocol. 

## Results

*Demographic and baseline characteristics of patients*

**Figure 1:**
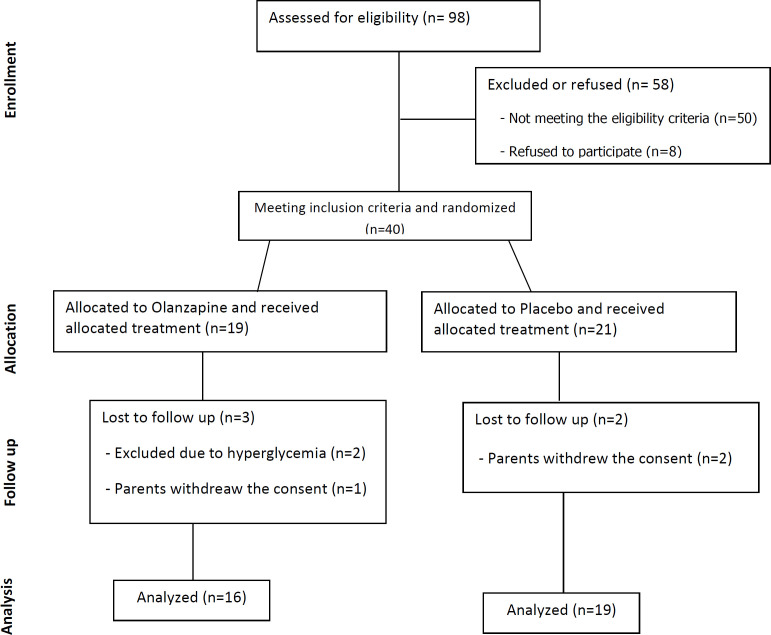
Consort flow diagram depicting the process of patient enrollment and interventions

A total of 98 patients were screened for eligibility. Forty patients who met the eligibility criteria were randomly assigned to receive placebo or olanzapine, in addition to standard treatment of ondansetron and dexamethasone. Five patients (10.6%) left the study and a final number of 35 patients completed the study (Figure 1). All baseline demographics and laboratory data including age, weight, gender, fasting blood glucose (FBS), prolactin, lipid profile, hepatic transaminases, and blood pressures except direct bilirubin, were similar in the olanzapine and placebo group. In both groups, direct bilirubin was within the normal limit (Table 1). 

*Analysis of chemotherapy-induced nausea and vomiting*

Among 19 patients taking ondansetron and dexamethasone with placebo, three patients (15.8%) developed vomiting based on the MAT scale: 1 patient with both acute and delayed vomiting and two patients had only acute vomiting. One patient (5.3%) experienced vomiting (delayed) according to the CTCAE scale. In the control group, based on the MAT scale, nausea was experienced in 2 (10.5%) cases: one case had acute nausea and one had delayed nausea. Using the CTCAE scale, 4 (21.1%) cases experienced nausea: 3 cases of acute nausea and 1 case of delayed nausea (Table 2). 

In the olanzapine group, 3 patients (18.8%) developed vomiting: 2 patients had acute vomiting and 1 patient had delayed vomiting. MAT and CTCAE scales yielded similar results in this regard (Table 2). Using the MAT scale, nausea was recorded for 6 patients (37.5%): 4 patients had acute nausea and 2 patients had delayed nausea. When using the CTCAE scale, nausea was recorded in 5 patients (31.3%): 3 patients had acute nausea and 2 patients had delayed nausea.

Complete response was observed in 84.2% of control patients and 68.8% of olanzapine patients. Statistical analysis did not show any significant difference (Table 2). The rates of acute or delayed vomiting were not significantly different between control and olanzapine groups using MAT or CTCAE emesis assessment (Table 2). Similarly, the onset of nausea was not significantly different between control and olanzapine groups using MAT or CTCAE scales (Table 2). 

*Safety analysis of olanzapine for the prevention of CINV in children*

Given the clinical and biochemical side effects profile of olanzapine, we evaluated the metabolic abnormalities and any possible side effects of intervention by comparing control and olanzapine groups (20). Comparing values of weight, FBS, prolactin, triglyceride, cholesterol and systolic and diastolic blood pressure, there was no significant difference between baseline values and values recorded after treatment in control or olanzapine groups (Table 3). In terms of other side effects, drowsiness (*P *= 0.036) and constipation (*P *= 0.056) were more frequently observed in the olanzapine group compared with the control group. Other adverse effects including back pain, insomnia, thirst, photophobia, stomach pain and loss of appetite were not significantly different (Table 4).

## Discussion

In this study, we assessed the efficacy of adding olanzapine to the standard regimen including ondansetron and dexamethasone, to prevent CINV in children receiving moderately emetogenic chemotherapy.

Olanzapine has been used to treat behavioral problems in the pediatric population. Studies have shown that three weeks of treatment with olanzapine is associated with increased blood pressure and metabolic derangements in the form of increased blood glucose, cholesterol and triglycerides (21). There was no report of extrapyramidal syndrome following three weeks of olanzapine use. Adverse effects with a shorter duration of olanzapine use have not been well-documented in the pediatric population. 

We observed complete response in the majority of patients receiving the standard protocol for CINV treatment. Adding olanzapine (0.14 mg/kg) to the standard regimen including ondansetron and dexamethasone was not associated with a significant beneficial effect in managing acute or delayed CINV. Regarding the safety, administration of olanzapine was associated with mild adverse effects including drowsiness and constipation. Our results also indicated that adherence to standard regimen does not prevent the CINV in all patients and further emphasizes that the standard protocol needs to be optimized to reach the perfection in full prophylaxis of CINV in children.

Management of CINV is an integral part of chronic cancer care and affects adherence to therapy and quality of life, especially in pediatric patients (22). Currently, three classes of drugs including 5-HT inhibitors, NK1 inhibitors and glucocorticoids are the main prophylactic modalities in CINV management (23). However, despite adherence to guidelines, up to 20-30% variability may be observed in clinical response due to individual risk factors and pharmacogenomic variations, leading to different hepatic metabolism and therapeutic outcomes (24). Thus, optimizing these protocols by adding other safe classes of medications is still an important subject of future research. 

It has been previously shown that olanzapine can increase the response rate of anti-emetic drugs in adult patients, especially in the management of delayed and breakthrough CINV (14, 25). Navari *et al. *showed that 10 mg olanzapine, in combination with dexamethasone, a 5-HT receptor inhibitor and NK1 inhibitor significantly improve the prevention of nausea and vomiting in patients undergoing cisplatin or cyclophosphamide-doxorubicin based chemotherapy, both among highly emetogenic chemotherapy agents. Accordingly, Wang *et al.* showed that using 5 mg olanzapine in combination with ondansetron and dexamethasone effectively improved the complete response rate in adult patients receiving cisplatin-based chemotherapy (26). In another study from Sudan performed on 131 patients, the olanzapine-containing regimen was superior in inducing complete response and nausea control in adult patients and its use was recommended in clinical practice due to efficacy and cost-effectiveness (27). 

In children, however, we lack strong evidence regarding the efficacy of olanzapine administration on improving the prophylaxis of CINV. A retrospective study provided preliminary evidence that olanzapine (0.1 mg/kg/dose), which was mainly prescribed due to inefficiency of CINV preventive protocols, induced complete control of vomiting in children (median age 13 years) undergoing chemotherapy with no serious safety concerns (28). Therefore, the use of olanzapine has been suggested in breakthrough control of CINV (16). Besides, a recent feasibility study on 15 pediatric patients of <18 years of age receiving 0.14 mg/kg/dose olanzapine, showed that eight patients had complete control of nausea following addition of olanzapine to their CINV prophylactic regimen, but 14 patients had nausea despite taking olanzapine (29). Given some degrees of efficacy a future trial focused on addition of olanzapine was recommended. The current study, although performed as a pilot study, showed that effect of olanzapine was more prominent on prophylaxis of vomiting than nausea in children. 

To the best of our knowledge, the current study is the first placebo-controlled blinded clinical trial to address the use of olanzapine in the pediatric population to prevent CINV. Our results demonstrated that adding olanzapine to the standard regimen including ondansetron and dexamethasone, did not significantly affect the management of acute or delayed CINV. We used two clinical scales of MAT and CTCAE to measure the frequency of CINV in pediatric patients following chemotherapy. Both scales concurred in lack of additive efficacy of olanzapine when compared to the standard regimen. Regarding the dose of olanzapine, unlike the retrospective study mentioned above, we used the dose of 0.14 mg/kg, referring to the dose used by Flank *et al.* which assumed that pharmacokinetics of olanzapine has shown to be similar in pediatrics and adults (28, 29). Our different findings might be likely due to the difference in the inherent response of prophylaxis *versus* treatment of acute or delayed vomiting. The emetogenic risk of the chemotherapies in our study was categorized as moderate according to the COG guidelines. However, this category consists of different chemotherapeutics with chances of emetogenicity ranging from 30% to 90% in the absence of CINV prophylaxis, which is a quite wide range. Therefore, it is possible that different chemotherapies that belong to this category may lead to varying efficiency outcomes of CINV preventive medications. Moreover, different chemotherapeutic agents with a moderate risk of emetogicity are administered with different dosing and duration of therapy, all of which may affect the efficacy of CINV prophylaxis when different study outcomes are directly compared. 

The administration of olanzapine was associated with mild adverse effects including drowsiness and constipation. These adverse reactions were tolerable and none of the participants discontinued the study due to side effects. 

**Table 1:** Demographic characteristics and laboratory values presented as median (interquartile range).

	**Placebo**	**Olanzapine**	***P*** **-value**
No of Cases	19	16	N/A
Age (Year)	10 (4-14)	8.5 (6-12)	0.803
Gender (M)	52.6%	81.3%	0.079
Baseline Weight (kg)	27 (20-57)	29 (22.5-58.5)	0.882
Baseline FBS	89 (81-98)	92.5 (88-99.5)	0.388
Baseline Prolactin	23 (17.1-36.2)	22.55 (11.3-31.9)	0.466
Baseline Triglyceride	97 (65-110)	87 (63.5-107.5)	0.631
Baseline Cholesterol	168 (146-184)	158.5 (139.5-180.5)	0.456
Baseline ALT	34 (15-72)	32 (25-66.5)	0.715
Baseline AST	24 (18-49)	28.5 (21.5-41.5)	0.508
Baseline Total Bilirubin	0.4 (0.4-0.6)	0.6 (0.4-0.6)	0.060
Baseline Direct Bilirubin	0.2 (0.2-0.3)	0.3 (0.2-0.4)	0.039
Baseline Systolic BP	110 (100-110)	110 (110-110)	0.127
Baseline Diastolic BP	70 (65-75)	71 (67.5-75)	0.626

Two groups did not differ in their baseline characteristics except for baseline direct bilirubin (*P*-values extracted by Mann-Whitney test). ALT: alanine aminotransferase; AST: aspartate aminotransferase; BP: blood pressure; FBS: fasting blood sugar; N/A: not applicable.

**Table 2:** Comparison of acute and delayed nausea and vomiting between placebo and olanzapine groups

	**Placebo (%)**	**Olanzapine (%)**	**Pearson Chi2**	***P*** **-value**
Acute Nausea (MAT)	1 (5.3%)	4 (25%)	2.9646	0.227
Acute Vomiting (MAT)	3 (15.8%)	2 (12.5%)	2.8975	0.575
Delayed Nausea (MAT)	1 (5.3%)	2 (12.5%)	3.2669	0.352
Delayed Vomiting (MAT)	1 (5.3%)	1 (6.3%)	2.0305	0.362
Acute Nausea (CTACE)	3 (15.8%)	3 (18.8%)	1.2625	0.532
Acute Vomiting (CTACE)	3 (15.8%)	2 (12.5%)	2.8975	0.575
Delayed Nausea (CTACE)	1 (5.3%)	2 (12.5%)	3.2669	0.195
Delayed Vomiting (CTACE)	1 (5.3%)	1 (6.3%)	2.0305	0.362
Complete Response (CR)	16 (84.2%)	11 (68.8%)	N/A	0.287

MAT: Multinational Association for Supportive Care in Cancer Anti-emesis Tool; CTCAE: Common Toxicity Criteria for Adverse Events; *P*-value extracted by Pearson Chi^2^ test, except complete response (CR) was analyzed by a test of proportions.

**Table 3:** Systolic and diastolic blood pressure, metabolic characteristics and hepatic transaminase status before and after the intervention

	**Placebo**			**Olanzapine**		
	**Baseline**	**After** **Treatment**	***P-v*** **alue**	**Baseline**	**After treatment**	***P*** **-value**
Weight (kg)	27 (20-57)	27 (20-58)	0.806	29 (22.5-58.5)	29.3 (23-58)	0.746
FBS	89 (81-98)	94 (82-114)	0.084	92.5 (88-99.5)	89 (82-99)	0.598
Prolactin	23 (17.1-36.2)	19.1 (13-26.1)	0.917	22.55 (11.3-31.9)	18.4 (10.6-26)	0.773
Triglyceride	97 (65-110)	95 (79-121)	0.676	87 (63.5-107.5)	92.5 (73-109)	0.500
Cholesterol	168 (146-184)	158 (142-193)	0.820	158.5 (139.5-180.5)	145.5 (131-171.5)	0.773
ALT	34 (15-72)	23 (14-42)	0.999	32 (25-66.5)	36 (25-64.5)	0.598
AST	24 (18-49)	18 (15-25)	1.000	28.5 (21.5-41.5)	31 (17.5-44)	0.895
Systolic BP	110 (100-110)	100 (100-110)	0.999	110 (110-110)	110 (105-110)	0.965
Diastolic BP	70 (65-75)	70 (65-75)	0.696	71 (67.5-75)	70 (65-70)	0.927

*P*-values calculated by Wilcoxon matched-pairs signed-ranks test. ALT: alanine aminotransferase; AST: aspartate aminotransferase; BP: blood pressure; FBS: fasting blood sugar.

**Table 4:** Adverse drug reactions following the use of placebo and olanzapine

**Side Effects (%)**	**Placebo**	**Olanzapine**	***P*** **-value**
Drowsiness	5 (26.3%)	9 (56.3%)	0.036
Constipation	0 (0%)	2 (12.5%)	0.056
Back pain	0 (0%)	1 (6.3%)	0.134
Insomnia	0 (0%)	2 (12.5%)	0.056
Thirst	1 (5.3%)	2 (12.5%)	0.223
Photophobia	0 (0%)	1 (6.3%)	0.134
Stomach pain	0 (0%)	1 (6.3%)	0.134
Appetite loss	1 (5.3%)	0 (0%)	0.824
Patients with any side effects	5 (26.3%)	9 (56.3%)	0.036

## Conclusion

According to this trial results, adding olanzapine to the standard regimen of CINV prophylaxis did not improve CINV prophylaxis in pediatric patients receiving moderately emetogenic chemotherapy.
